# Genetic parameters of infectious bovine keratoconjunctivitis and its relationship with weight and parasite infestations in Australian tropical *Bos taurus* cattle

**DOI:** 10.1186/1297-9686-44-22

**Published:** 2012-07-27

**Authors:** Abdirahman A Ali, Christopher J O’Neill, Peter C Thomson, Haja N Kadarmideen

**Affiliations:** 1 ReproGen – Animal Bioscience Group, Faculty of Veterinary Science, University of Sydney, Camden, NSW, 2570, Australia; 2 Section of Genetics and Bioinformatics, Department of Veterinary Clinical and Animal Sciences, Faculty of Health and Medical Sciences, University of Copenhagen, 1870, Frederiksberg C, Copenhagen, Denmark; 3 CSIRO Animal, Food and Health Sciences, Australian Tropical Science and Innovation Precinct, James Cook University, PMB PO Aitkenvale, Townsville, QLD, 4814, Australia

## Abstract

**Background:**

Infectious bovine keratoconjunctivitis (IBK) or ‘pinkeye’ is an economically important ocular disease that significantly impacts animal performance. Genetic parameters for IBK infection and its genetic and phenotypic correlations with cattle tick counts, number of helminth (unspecified species) eggs per gram of faeces and growth traits in Australian tropically adapted *Bos taurus* cattle were estimated.

**Methods:**

Animals were clinically examined for the presence of IBK infection before and after weaning when the calves were 3 to 6 months and 15 to 18 months old, respectively and were also recorded for tick counts, helminth eggs counts as an indicator of intestinal parasites and live weights at several ages including 18 months.

**Results:**

Negative genetic correlations were estimated between IBK incidence and weight traits for animals in pre-weaning and post-weaning datasets. Genetic correlations among weight measurements were positive, with moderate to high values. Genetic correlations of IBK incidence with tick counts were positive for the pre-weaning and negative for the post-weaning datasets but negative with helminth eggs counts for the pre-weaning dataset and slightly positive for the post-weaning dataset. Genetic correlations between tick and helminth eggs counts were moderate and positive for both datasets. Phenotypic correlations of IBK incidence with helminth eggs per gram of faeces were moderate and positive for both datasets, but were close to zero for both datasets with tick counts.

**Conclusions:**

Our results suggest that genetic selection against IBK incidence in tropical cattle is feasible and that calves genetically prone to acquire IBK infection could also be genetically prone to have a slower growth. The positive genetic correlations among weight traits and between tick and helminth eggs counts suggest that they are controlled by common genes (with pleiotropic effects). Genetic correlations between IBK incidence and tick and helminth egg counts were moderate and opposite between pre-weaning and post-weaning datasets, suggesting that the environmental and (or) maternal effects differ between these two growth phases. This preliminary study provides estimated genetic parameters for IBK incidence, which could be used to design selection and breeding programs for tropical adaptation in beef cattle.

## Background

**Figure 1:**
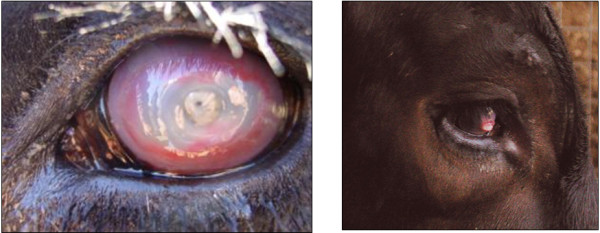
**Examples of infectious bovine keratoconjuctivitis in cattle.** Left: a severe IBK case showing complete corneal opacity (score 6); right: a healing stage showing central cornea from 3 to 6 mm in diameter (score 3). Photographs courtesy of Dr E. Casas.

Cattle reared in tropical northern Australia are exposed to varying levels of environmental challenges, including high ambient temperature and high incidence of infection by a variety of pathogens and ecto- and endo-parasites. Over the past 40 years, a number of crossbreeding experiments have been conducted to examine genetic and environmental aspects of cattle performance under the influence of such tropical environmental stressors. Since the 1950’s, one of the most important animal breeding experiments in Australia has been the development of a cross between Hereford and Shorthorn cattle (hereafter named the HS line), which is commercialised under the name ‘Adaptaur’; bulls from the HS line were released to the beef industry to provide genetic material. The HS line remains a ‘closed’ line of pure British *Bos taurus* breeds with unique characteristics [1]. Although its level of genetic diversity is relatively low [2], it has been successfully selected for both production traits (i.e. high post-weaning growth) and for increased resistance to cattle ticks, while enduring various environmental stressors such as ecto-parasites and endo-parasites, heat, humidity and seasonal variation in pasture quality [3]. A combination of natural and artificial selection has resulted in a breed with a relatively high level of acquired immunity to cattle tick infestations [4]. In Australia, there have been several studies on HS composites [5,6] and other tropical beef cattle [7,8] that have investigated genetic and phenotypic parameters for growth, reproduction, external and internal parasite tolerance. However, there is a lack of information on the genetic and phenotypic parameters for infectious bovine keratoconjunctivitis (IBK) in tropical cattle composites. IBK, commonly known as ‘pinkeye’, is an inflammatory bacterial infection that affects the eyes of cattle. It is characterised by inflammation of the conjunctiva, ulceration of the cornea, excessive tearing, and in severe cases, perforation of the cornea which can lead to permanent blindness (Figure 1). In the 1980’s, an Australian postal survey recorded that 81% of cattle owners reported the occurrence of IBK in their herds and 75% observed a reduction in weight gain of the affected cattle [9]. Although this survey is outdated, IBK remains a serious problem for livestock industries, with an estimated annual incidence of 10% that costs over $AU 23 million in cattle production losses and management [10]. In the United States, more than 10 million calves are affected by IBK annually, with an estimated economic loss of more than $US 150 million [11]. Hence, cattle producers are keen to reduce the incidence of IBK by selective breeding. Although improving herd management, treatment or vaccination may provide a short-term solution, genetic selection is an alternative long-term solution to control IBK incidence in cattle.

Analysis of health records of 45 497 animals from purebred and crossbred *Bos taurus* and tropically adapted breeds, i.e. mainly from Brahman, Boran and Tuli cattle populations, estimated heritability for IBK incidence ranging from 0.00 to 0.28 [12].

A significant between-breed difference in IBK incidence has been reported [12,13], with the purebred Hereford breed being the most susceptible compared to other pure and composite breeds. Thus, sufficient genetic variation for resistance to IBK exists in these breeds to consider genetic selection. To date, genetic parameters for resistance to IBK in Australian tropical composite breeds have not been reported. Thus, the main objectives of our study were to estimate genetic parameters and breeding values for resistance to IBK of a HS composite population at different stages of cattle growth. Results will enable northern Australian cattle producers to select animals with high genetic merit for resistance to IBK. However, before selection decisions are made, it is important to quantify possible genetic antagonisms between IBK and growth traits. For example, in dairy cattle, the increased genetic merit for milk yield is associated with a decline in average fertility and health (i.e. mastitis, claw and foot disorders) [14-16]. Therefore, another objective of our study was to estimate the genetic and phenotypic correlations between IBK and growth and parasite infestation traits by including these in a multi-trait genetic parameter estimation program. These multi-trait settings make use of economic weights more optimal in a total genetic merit index.

## Methods

### Animals

Genetic and phenotypic parameters for IBK, weight measurements at different ages, helminth egg count and cattle tick count were estimated in an HS composite cattle population. A detailed history of the tropically adapted Australian HS composite cattle population has been reported elsewhere [4]. Briefly, the herd was developed by the Commonwealth Scientific and Industrial Research Organisation (CSIRO) during the 1950s at the National Cattle Breeding Station, ‘Belmont’, latitude 23.22 °S longitude 150.38 °E, near Rockhampton, Queensland, Australia, and was a nominal composition of 50% Hereford and 50% Shorthorn. Animals were born from October to December each year and weaned around March or April of the following year from 1973 to 1998. These animals were reared under similar stressful environmental conditions, which included high ambient temperatures and humidity, high IBK incidence and gastro-intestinal and external parasites [17].

### Phenotypic data for IBK

Data on both the incidence and severity of IBK infection were collected from 1973 to 1997. Calves were observed for clinical signs of IBK at two stages of development, first, when they were 3 to 6 months old (‘pre-weaning’ time point) and second, when they were15 to 18 months old (‘post-weaning’ time point). The pre-weaning and post-weaning assessments involved 932 calves (403 males and 529 females) and 1 084 adult animals (514 males and 570 females), respectively. The pedigree of these animals was traced back to four generations and contained 3 360 animals sired by 168 sires (average of 20 progeny per sire) from 990 dams.

Animals were restrained in a head-bail located under an open-sided shelter and clinical examinations were performed by trained staff. Each eye of every animal was scored on a 1 to 6 scale, with 1 = cornea showing no clinical signs of ulceration or opacity (this score was used as a base line or reference); 2 = healing central corneal scar less than 3 mm in diameter, opaque but without active vessels; 3 = healing central cornea from 3 to 6 mm in diameter, opaque but without active vessels; 4 = healing central cornea scar greater than 6 mm diameter, opaque but without active vessels; 5 = dense corneal opacity, central opaque area less than 5 mm diameter with complete vascular ring; and 6 = complete dense corneal opacity, eye often with complete blindness. IBK score of the left and right eye were summed for each animal, ranging from a score of 2 for healthy eyes and scores 3 to12 for increasingly diseased eyes. Sizes of the lesions were qualitative estimates. The system of scoring was an attempt to classify the infection according to its severity and whether the lesion was active (and hence of direct effect at the time of scoring) or an inactive scar from a previous infection. No attempt was made to isolate the causal organism.

### Phenotypic data for tick counts

Cattle tick (*Rhipicephalus microplus*) counts (TKC) were performed once every 21 days during the tick season, generally late autumn to early winter and/or spring to early summer for the years 1979 and 1983 through 1997. TKC counts were repeated three times per animal and consisted of counting the number of engorging female ticks 4.5 to 8.0 mm long on one side of every animal following field infestation [18].

Mean of these three TKC counts was used for the assessment of tick resistance. A count was considered usable if the mean count for that month was equal or greater than 20 ticks per animal side to allow for sufficient variation. The TKC counts were recorded on 875 animals (430 males and 445 females) from weaning (approximately 6 months) to 18 months of age. The animals originated from 57 sires with progeny group sizes ranging from 1 to 64, with an average of 14 animals per sire.

### Phenotypic data for helminth egg counts

The number of helminth (unspecified species) eggs per gram (EPG) of faeces were counted on a fresh faecal sample using a McMaster slide under a microscope at 100 × magnification [19].

The EPG counts were assessed as the mean of the first three EPG counts recorded on each animal after weaning (approx. 6 to 9 months of age) during the period between 1987 and 1998. Those EPG counts were taken three weeks apart during parasite season (from June to July). A total of 727 animals (363 males and 364 females) with EPG counts were available. These animals were offspring of 53 sires (average 14 progeny per sire, ranging from 1 to 70).

### Phenotypic data for growth

Four live weight measurements were taken at several ages from birth to 18 months for the period between 1978 and 1998. At birth, calves were individually identified and weighed (birth weight = BWT, kg). Weights for weaning (WWT, kg), yearling (YWT, kg) and final weight (FWT, kg) were recorded when animals were approximately 6, 12 and 18 months of age, respectively. A total 1 298 animals (636 males and 662 females) with weight measurements were available in this study. Animals were progeny of 77 sires (average 17 progeny per sire, ranging from 1 to 70).

### Statistical analyses

Data provided for the present study had previously been recorded as the sum of the left and right IBK scores for each animal, thus, we could not perform ordinal regression (multiple thresholds) analyses, which would otherwise have been the most appropriate analysis. Consequently we converted the summed scores to a binary (0/1) trait, setting score 2 equal to 0 (i.e., IBK absent) and scores 3 to 12 equal to 1 (IBK present). Most health and reproduction traits are usually given as all-or-none (0/1), and it is often assumed that 0/1 traits have an underlying continuous distribution [20]. There are several examples in the literature where ordinal multiple scores of lesions or disorders in animals have been converted to a simple binary form, e.g. for osteochondral bone disease in pigs [21,22] and for hip dysplasia in dogs [23].

First, data (phenotypes and fixed effects terms) exploration was performed for each trait using numerical and graphical descriptive analyses (i.e., outliers, distributions and relationships). This has revealed that TKC and EPG counts were not normally distributed. Thus, consistent with previous studies [5,6], counts were transformed using log_10_ (mean TKC + 1) for mean TKC counts and a cube-root transformation for mean EPG counts. Quantile-quantile plots indicated that these transformations produced residuals that approached normality (data not shown). All genetic parameter estimates for TKC and EPG counts presented here were based on the transformed scale. Then, significance of environmental fixed effects was assessed (using a statistical significance threshold of *P* < 0.05) by fitting linear regression models using the **lm** function for TKC, EPG and weight traits, and the **glm** function for IBK binary in the R software package [24]. The effects included in the models were age in number of days as a covariate, year when traits were recorded, and sex of the calf for IBK, TKC and EPG traits. For weight traits, the previous lactation status of the dam was included as an additional fixed effect (1 = maiden, 2 = having lactated in the previous year, 3 = dry non-pregnant cow in the previous year, and 4 = dry, cow calved in the previous year but the calf did not survive).

### Variance components estimation

A univariate animal model was fitted to the data using ASReml [25] to estimate variance components, heritability and breeding values for each trait considered. The basic linear mixed model notation was:

y=Xb+Za+e

where **y** = vector of phenotypes (TKC, EPG or weight traits), **b** = vector of fixed effects included in the model, as described previously, **X** = incidence matrix for fixed effects, **a** = vector of random animal effects, **Z** = incidence matrix of random animal effects and **e** = random residual effects.

The animal and residual terms were assumed to be $\text{a}\sim \text{N}(0\text{,}\;A\sigma_{a}^{2})\text{;}\;\text{e}\sim \text{N}(0\text{,}\;I\sigma_{e}^{2})$ where $\sigma_{a}^{2}$ and $\sigma_{e}^{2}$ are variances of random animal genetic effect and random residual error effect, respectively. **A** is a numerator relationship matrix across all animals inferred from the pedigree (n = 3 360), and **I** is an identity matrix with an order equal to the number of observations in **y**.

Binary IBK trait was analyzed using a generalised linear mixed model (GLMM) with the logit link function. The model in matrix notation was:

logit(π)=Xβ+Za

where **π** = vector of probabilities of IBK present (= 1). The fixed and random effects were the same as used in the linear mixed models. Random effects in the model were assumed to be from multivariate normal distributions as described previously.

Heritability on underlying scale was calculated as:

$$h^{2}=\frac{\sigma_{a}^{2}}{\left(\sigma_{a}^{2}+\pi^{2}/3\right)}$$

where $\sigma_{a}^{2}$ is the estimate of the additive genetic variance and π^2^/3 is the liability variance scale for the binomial distribution, where π^2^/3 ~ 3.28987.

### Bivariate model for estimation of genetic and phenotypic correlation

In order to estimate genetic and phenotypic relationships among the traits considered, the individual datasets for TKC, EPG and weight were merged with both the IBK pre-weaning dataset and the IBK post-weaning dataset, resulting in a pre-weaning dataset with 535 animals and a post-weaning dataset with 650 animals. Although our intention was to fit a multi-trait model that analysed all traits simultaneously, this was not feasible due to computational limitations; therefore, in order to estimate genetic and phenotypic correlations, bivariate animal models were fitted for various combinations of traits.

The bivariate animal model can be represented as follows:

$$\left[\begin{array}{l} y_{1}\\ y_{2} \end{array}\right]=\left[\begin{array}{ll} X_{1} & 0\\ 0 & X_{2} \end{array}\right]\;\left[\begin{array}{l} b_{1}\\ b_{2} \end{array}\right]+\left[\begin{array}{ll} Z_{1} & 0\\ 0 & Z_{2} \end{array}\right]\;\left[\begin{array}{l} a_{1}\\ a_{2} \end{array}\right]+\left[\begin{array}{l} e_{1}\\ e_{2} \end{array}\right]$$

where **y**_*i*_ = vector of observations for the *i*^th^ trait (*i* = 1 or 2), **b**_*i*_ = vector of fixed effects for the *i*^th^ trait (*i* = 1 or 2), **a**_*i*_ = vector of random animal effects for the *i*^th^ trait (*i* = 1 or 2), **e**_*i*_ = vector of random residual effects for the *i*^th^ trait (i = 1 or 2), **X**_*i*_ = incidence matrices relating records of the *i*^th^ trait to fixed effects (*i* = 1 or 2), **Z**_*i*_ = incidence matrices relating records of the *i*^th^ trait to random animal effects (*i* = 1 or 2).

The expectations and variance were

$$\text{E}\;\left[\begin{array}{l} y_{1}\\ y_{2} \end{array}\right]=\left[\begin{array}{ll} X_{1} & 0\\ 0 & X_{2} \end{array}\right]\;\left[\begin{array}{l} b_{1}\\ b_{2} \end{array}\right]\;$$

and

$$\text{Var}\;\left[\begin{array}{l} a_{1}\\ a_{2}\\ e_{1}\\ e_{2} \end{array}\right]=\left[\begin{array}{llll} A\;\sigma_{a1}^{2} & A\;\sigma_{a12} & 0 & 0\\ A\;\sigma_{a21} & A\;\sigma_{a2}^{2} & 0 & 0\\ 0 & 0 & I\sigma_{e1}^{2} & I\sigma_{e12}\\ 0 & 0 & I\;\sigma_{e21} & I\sigma_{e2}^{2} \end{array}\right]$$

where$\sigma_{a1}^{2}\;\text{and}\;\sigma_{a2}^{2}$ are additive genetic variances for traits 1 and 2, $\sigma_{a12}=\sigma_{a21}$ is the additive genetic covariance between traits 1 and 2,$\sigma_{e1}^{2}\;\text{and}\;\sigma_{e2}^{2}$ are residual variances for traits 1 and 2, $\sigma_{e12\;}=\sigma_{e21}$ is the residual covariance between traits 1 and 2,

**A** and **I** are as described previously, **a** and **e** were assumed to be normally distributed with a mean of zero and (co)variances as specified above.

Using the (co-)variance component estimates, genetic correlations were calculated as

rg=σa12σa12×σa2212.

and phenotypic correlations as

rp=σa12+σe12(σa12+σe12)×(σa22+σe22)12.

## Results and discussion

### Descriptive statistics

Resistance of the tropically adapted HS line to tropical parasites is well documented [5]. In this work, we have investigated its resistance to IBK. Summary statistics for the traits considered are presented in Table 1. IBK status was scored on 932 calves before weaning (aged 3 to 6 months) and on 1 084 calves after weaning (aged 6 to 18 months). Mean IBK incidences were 0.34 and 0.21 for pre-weaning and post-weaning calves, respectively. The higher pre-weaning IBK incidence is consistent with the results of a previous study on purebred Herefords [26]. The average raw (untransformed) TKC and EPG counts were 53.3 and 566.1. Coefficients of variation for the traits measured ranged from 13.6 to 88.6%, with the raw (untransformed) data for TKC counts having the highest relative variability (see Table 1).

**Table 1:** Summary statistics for the traits studied

**Trait**	**Number of animals**	**Mean**	**SD**^**1**^	**Min**^**2**^	**Max**^**3**^	**CV**^**4**^
IBK pre-weaning	932	0.3†	0.5	0	1	-
IBK post-weaning	1 084	0.2†	0.4	0	1	-
Raw mean tick count	875	53.3	33.5	20.0	211.4	62.9
Transformed mean tick count ^5^	875	3.8	0.5	3.0	5.4	13.8
Raw mean EPG	727	566.1	501.3	5.0	3 115.0	88.6
Transformed mean EPG^6^	727	7.6	2.4	1.7	14.6	32.0
Birth weight	1 276	32.6	4.4	13.0	56.0	13.6
Weaning weight	1 198	151.0	27.6	67.0	260.0	18.3
Yearling weight	1 074	172.5	34.0	80.0	313.0	19.7
Final weight	1 038	256.4	41.6	106.0	405.0	16.2

†Infectious bovine keratoconjunctivitis (IBK) incidence for pre-weaning and post-weaning animals; ^1^*SD* = standard deviation; ^2^Minimum; ^3^Maximum; ^4^*CV* = coefficient of variation (%); ^5^log_10_ (mean tick count + 1); ^6^cube root mean worm egg counts transformed values.

**Figure 2:**
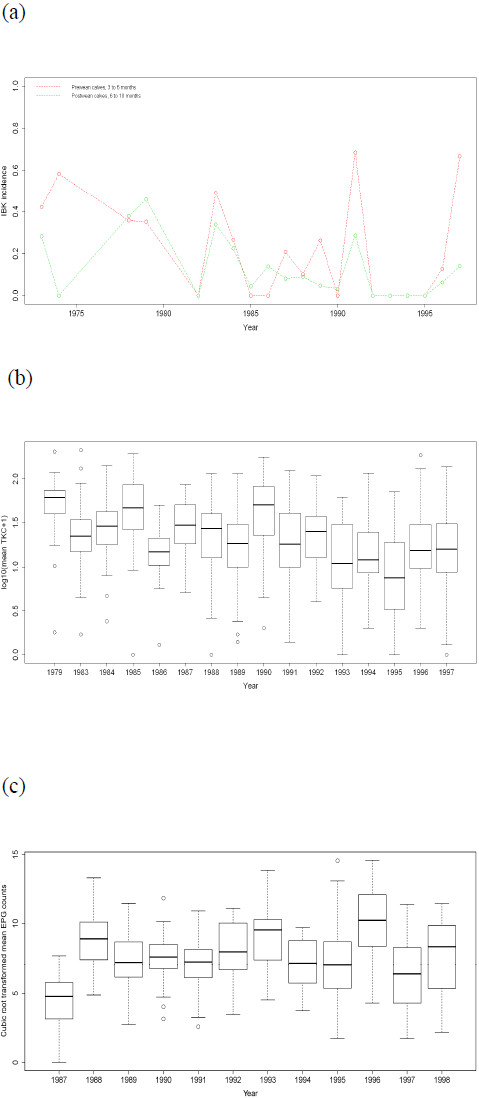
**Variation in disease traits across years of tropically adapted cattle.** (**a**) IBK incidence between 1973 to 1997 years for pre-weaning (red dots) and post-weaning (green dots); (**b**) log_10_ transformed mean tick count for 1979, 1983 to 1997; (**c**) cube-root transformed mean worm egg count for 1987 to 1998.

IBK incidence varied across years (see Figure 2a), with a peak in 1991. Pre-weaning calves exhibited a higher IBK incidence than post-weaning calves in the years during which IBK occurrence increased i.e. 1973–1974, 1983–1984, 1991 and 1997. TKC and EPG counts varied across years (see Figures 2b-c, respectively), with large median counts observed in 1979, 1985 and 1990 for TKC counts, and in 1988, 1993 and 1996 for EPG counts. Analyses of fixed effects showed that year when traits were recorded and age (in days) were statistically significant (*P* < 0.05) for all traits. For the weight traits, the fixed effects of sex and previous lactation status of the dams were also significant (*P* < 0.05).

### Genetic parameter estimates

#### Heritabilities

Estimates of heritabilities and their standard errors estimates were obtained using univariate (or single trait) analyses and are presented in Table 2. Estimates of heritability for IBK were small to moderate for both pre-weaning and post-weaning calves (0.17 ± 0.06 to 0.19 ± 0.03, respectively). These estimates were consistent with a previous study that reported a moderate direct heritability of 0.20 ± 0.06 for IBK incidence in *Bos indicus* × *Bos taurus* crossbred calves [12]. They examined a large dataset with health records over 20 years for IBK incidence in calves of multiple breeds. Estimates of heritability ranged from 0.00 to 0.28, with the largest estimates for Hereford (*h*^*2*^ = 0.28), composite ‘MARC III’ (*h*^*2*^ = 0.26) and Angus (*h*^*2*^ = 0.25) breeds. The heritability estimate for log transformed TKC counts was low to moderate (*h*^2^ = 0.20 ± 0.12). This estimate is comparable to those reported in several Australian studies on Brahman and tropical composite populations (0.13 ± 0.03 to 0.34 ± 0.03) [5,6,8]. A study on South African Bonsmara cattle reported heritability estimates of 0.17 ± 0.05 for Box-Cox transformed mean tick counts [27]. However, the current heritability estimate of TKC is lower than that reported in Belmont Red cattle (0.42) in Australia [28].

**Table 2:** Heritabilities and standard errors estimated with the univariate model

**Trait**^**1**^	**Heritability**	**Standard error**
IBK before weaning	0.17*	0.03
IBK after weaning	0.19*	0.06
TKC	0.20	0.12
EPG	0.41	0.09
Birth weight	0.60	0.09
Weaning weight	0.47	0.09
Yearling weight	0.38	0.09
Final weight	0.35	0.10

^1^*IBK* = infectious bovine keratoconjuctivitis incidence; *TKC* = log_10_ (mean tick count + 1); *EPG* = cube-root mean worm egg counts, weaning weight at 6 months of age, yearling weight at 12 months of age and final weight at 18 months of age; *heritability estimate based on liability scale for binomial distribution.

Our estimate of the heritability of cube-root transformed EPG counts was moderate (*h*^2^ = 0.41 ± 0.09) and similar to that reported in Brahman heifers (0.40 ± 0.12) [8] and in several Australian studies (0.33 – 0.44) [5,6]. A study on Angus cattle under a natural challenge situation in New Zealand using a log_10_ (faecal count + 100) transformation reported a heritability of 0.32 [29].

In ruminants, immune response varies with the age of the host, the duration of parasite exposure, the number of parasites (dosage) and the nutrition status of the host [30,31]. In our study, differences in heritability estimates for the parasite-resistance traits may be due to differences in these factors and/or different populations, season effects, and models used.

Heritability estimates ranged from 0.35 ± 0.10 to 0.60 ± 0.09 for weight traits (Table 2). Estimates were 0.60, 0.47, 0.38 and 0.35 for BWT, WWT, YWT and FWT, with standard errors ranging from 0.09 to 0.10. Our estimates were slightly higher than those reported in Australian Angus cattle [32] but since maternal genetic and permanent environmental effects were not included, the estimates may be biased upward.

#### Estimated breeding values for IBK

**Figure 3:**
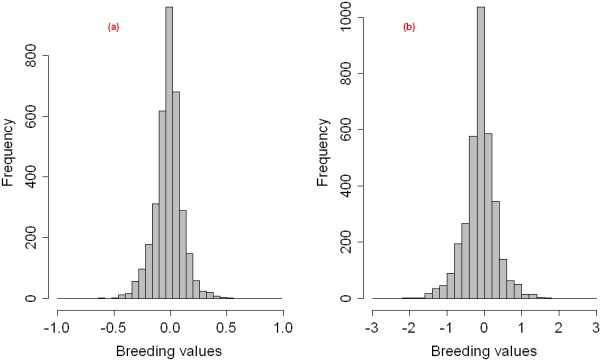
**Distribution of breeding values of IBK infection.** (**a**) breeding values for pre-weaning animals; (**b**) breeding values for post-weaning animals.

Distributions of estimated breeding values (EBV) for IBK pre-weaning and post-weaning datasets are presented in Figure 3. The extremes of each distribution show a small number of animals with either high or low EBV. The EBV has a narrower range for pre-weaning calves (i.e. from −0.5 to 0.5) than for post-weaning calves (from −2.0 to 2.0).

### Genetic and phenotypic correlations

Genetic and phenotypic correlations and their standard errors for different combinations of traits are presented in Tables 3 and 4 for the pre-weaning and post-weaning datasets, respectively. Genetic correlations between IBK and different weight measurements were all negative and ranged from −0.10 to -0.50 (s.e.: 0.15 to 0.30) for both datasets, except the correlation between IBK and BWT, which was close to zero for the post-weaning dataset. The negative sign of these genetic correlations indicates that animals that are genetically prone to acquiring IBK infection are also genetically prone to slower growth/weight gain.

**Table 3:** **Genetic**^1 ^**and phenotypic**^2 ^**correlations for pre-weaning dataset**

**Trait**^**3**^	**IBK**	**BWT**	**WWT**	**YWT**	**FWT**	**EPG**	**TKC**
IBK		−0.27 (0.24)	−0.12 (0.24)	−0.15 (0.23)	−0.10 (0.30)	−0.46 (0.26)	0.29 (0.27)
BWT	0.00 (0.05)		0.77 (0.10)	0.84 (0.12)	0.85 (0.09)	0.32 (0.20)	−0.03 (0.25)
WWT	0.00 (0.04)	0.50 (0.05)		0.96 (0.04)	0.78 (0.09)	0.22 (0.19)	0.18 (0.24)
YWT	−0.05 (0.05)	0.47 (0.05)	0.79 (0.02)		0.49 (0.14)	0.22 (0.25)	0.15 (0.32)
FWT	−0.16 (0.04)	0.48 (0.05)	0.68 (0.03)	0.65 (0.03)		0.23 (0.22)	0.09 (0.28)
EPG	−0.12 (0.05)	0.01 (0.06)	−0.04 (0.06)	−0.11 (0.06)	−0.05 (0.06)		0.33 (0.23)
TKC	0.06 (0.04)	0.02 (0.06)	−0.03 (0.06)	−0.16 (0.05)	−0.22 (0.05)	0.08 (0.06)	

^1^genetic correlations above diagonal; ^2^phenotypic correlations below diagonal; ^3^*IBK* = infectious bovine keratoconjuctivitis (0/1); *BWT* = birth weight (kg); *WWT* = weaning weight (kg); *YWT* = yearling weight (kg); *FWT* = final weight (kg); *EPG* = cube root transformed mean helminth egg count; *TKC* = log_10_ (mean tick count + 1); standard errors are in parentheses.

**Table 4:** **Genetic**^1 ^**and phenotypic**^2 ^**correlations for post-weaning dataset**

**Trait**^**3**^	**IBK**	**BWT**	**WWT**	**YWT**	**FWT**	**EPG**	**TKC**
IBK		0.02 (0.19)	−0.49 (0.15)	−0.50 (0.17)	−0.40 (0.19)	0.10 (0.21)	−0.14 (0.23)
BWT	0.10 (0.05)		0.49 (0.12)	0.50 (0.14)	0.69 (0.13)	0.11 (0.17)	−0.06 (0.19)
WWT	−0.53 (0.03)	0.34 (0.05)		0.91 (0.04)	0.74 (0.09)	0.03 (0.18)	0.17 (0.21)
YWT	−0. 50 (0.04)	0.33 (0.04)	0.84 (0.01)		0.97 (0.04)	0.03 (0.20)	−0.08 (0.23)
FWT	−0.42 (0.04)	0.35 (0.04)	0.71 (0.02)	0.79 (0.01)		0.13 (0.21)	−0.04 (0.24)
EPG	0.11 (0.04)	0.00 (0.05)	−0.10 (0.04)	−0.16 (0.05)	−0.08 (0.05)		0.31 (0.20)
TKC	0.03 (0.05)	0.03 (0.05)	0.02 (0.05)	−0.11 (0.04)	−0.18 (0.04)	0.05 (0.04)	

^1^genetic correlations above diagonal; ^2^phenotypic correlations below diagonal; ^3^*IBK* = infectious bovine keratoconjuctivitis (0/1); *BWT* = birth weight (kg); *WWT* = weaning weight (kg); *YWT* = yearling weight (kg); *FWT* = final weight (kg); *EPG* = cube root transformed mean helminth egg count; *TKC* = log_10_ (mean tick count + 1); standard errors are in parentheses.

For the pre-weaning dataset, the phenotypic correlation between IBK and FWT was −0.16 ± 0.04 but close to zero for all other weight traits (BWT, WWT and YWT) (Table 3). For the post-weaning dataset estimates of phenotypic correlations were negative between IBK and weight measurements, ranging from −0.42 ± 0.04 to -0.53 ± 0.03, except between IBK and BWT (0.10 ± 0.05) (Table 4). In general, calves with IBK appear to have a lower weight, which is consistent with previous studies [33].

Estimates of genetic correlations between IBK and TKC counts were 0.29 ± 0.27 and −0.14 ± 0.23 for the pre-weaning and post-weaning datasets (Table 3 and Table 4). Genetic correlations between IBK and EPG counts were negative at −0.46 ± 0.26 for the pre-weaning dataset The direction of genetic correlations of IBK with TKC and EPG counts were opposite between the two datasets. This may be due to the fact that the calves’ pre-weaning environment is different from the post-weaning environment, including with regard to maternal effects. To our knowledge, genetic relationships between IBK and TKC or EPG counts have not been previously reported.

Genetic correlations between TKC and EPG counts were positive in pre-weaning and post-weaning datasets (0.33 ± 0.23 and 0.31 ± 0.20, Tables 3 and 4). Such positive correlations suggest that pleiotropic genes may be involved in resistance to ecto-parasites and endo-parasites in this cattle population and that cattle that have developed tolerance/resistance to ticks through natural or artificial selection can also acquire tolerance/resistance to parasite infestation. The phenotypic correlations between TKC, EPG and IBK were low for both datasets.

Genetic correlations among weight traits were highly positive and significant for both datasets and ranged from 0.49 to 0.97 (Tables 3 and 4). These estimates were in line with reports from [34]. The positive genetic correlations observed between birth weight and other weight traits is not advantageous since it could lead to calving difficulty (dystocia) and decrease calf survival and welfare of the cow.

Tables 3 and 4 also present genetic parameters for environments with varying levels of environmental stress. For instance, HS calves born in north-east Queensland move from a benign uterine environment to a relatively protected maternal pre-weaning environment and then to a tropically stressful post-weaning environment. For a breed that has evolved in a temperate environment, this represents an extreme contrast in environmental stress. In the pre-weaning environment, the genetic correlations between IBK and the growth traits were consistently small and negative (Table 3), whereas in the post-weaning environment, after removal of the maternal influence on the calves, the genetic correlations are more negative, except with BWT (uterine environment), (Table 4). Similarly, the genetic correlations between the parasite traits and the growth traits all show lower estimates for the pre-weaning dataset compared to the post-weaning dataset (Tables 3 and 4). Estimates of the heritability of growth also differ depending on the level of environmental stress. The heritability estimates for BWT and WWT (uterine and maternal environment, respectively) are higher than, and in some cases almost double the estimates for YWT and FWT (see Table 2). The finding that estimates of heritability are lower in more environmentally stressful environments has been documented previously [35] and studies of Herefords from the semi-arid environment of Arizona, USA, [36,37] have also found that genetic parameters vary when estimated in “good’ versus “poor” environments. In an optimal environment, identifying sires with the optimal growth “resource allocation factor” [37] is straightforward but in a variable stressful environment, animal growth is more complex and must consider both inherent growth (inherent metabolic rate) and fitness (resistance to IBK and parasite infection); hence, a lower heritability for growth and modified genetic correlations in a stressful environment is expected.

Although the traditional quantitative genetic approach used here has assisted us to understand the adaptation of cattle in tropical climates and their ability to deal with environmental stressors, the true value of this information is in identifying genetic architecture affecting adaptation/fitness. The ultimate goal of such studies would be to provide information about molecular markers that could be used in marker-assisted programs and lead to the implementation of selection programs for the simultaneous improvement of growth and tropical adaptation/fitness.

## Conclusions

Genetic parameter estimates for infectious bovine keratoconjunctivitis (IBK), also known as ‘pinkeye’, and its genetic and phenotypic correlations with growth and ecto-and endo-parasite traits were investigated. IBK infection is a heritable trait, although its heritability is low to moderate (0.17 ± 0.03 to 0.19 ± 0.06) for pre-weaning and post-weaning calves from Australian tropically adapted *Bos taurus* cattle. Small to moderate negative genetic correlations between IBK and different weight measurements suggest that the animals that are genetically prone to acquire IBK infection may also be genetically prone to gain weight more slowly. The genetic correlations between IBK and TKC and between IBK and EPG counts display opposite values between pre-weaning and post-weaning datasets, suggesting differences in (maternal) environment between the two stages. However, more accurate estimates are needed; hence further data collection and pedigree recording on IBK, TKC and EPG is recommended. Nevertheless, our estimates of genetic parameters for IBK indicate that there is sufficient genetic variation in beef cattle to design selection and breeding programs for tropical adaptation of these breeds. Recording IBK cases on a routine basis and at a national level would allow inclusion of IBK resistance in the multi-trait breeding goal for the Australian beef industry, as well as in other countries, and would help address animal welfare and health issues.

## Competing interests

The authors declare that they have no competing interests.

## Authors’ contributions

AAA conducted data editing, analysis and wrote the first draft of this manuscript. HNK was the project leader and supervisor and contributed to the design of the statistical models and analytical methods and to the preparation of the early and final manuscript. PCT contributed to the statistical models and the preparation of the final manuscript. CJO was the experimental project leader and contributed to the preparation of the final manuscript. All authors read and approved the final manuscript.
